# Supplementary material for: Microtubule number and length determine cellular shape and function in Plasmodium
Source: EMBO J. 2019 May 24;38(15):e100984. doi: 10.15252/embj.2018100984 (PMC6669926; doi:10.15252/embj.2018100984)
Supplement: Supplementary file 1 — Appendix [file EMBJ-38-e100984-s001.pdf]

Appendix.

Microtubule number and length determine cellular shape and function in *Plasmodium*

Benjamin Spreng[1], Hannah Fleckenstein[1*], Patrick Kübler[1*], Claudia Di Biagio[1*], Madlen Benz[1*], Pintu Patra[2*], Ulrich S. Schwarz[2], Marek Cyrklaff[1] and Friedrich Frischknecht[1,#]

Table of contents

1. Appendix Supplementary Figures 1- 9 and figure legends
2. Appendix Table S1 – Primers
    a. Generation of $\alpha 1^{cm\&\Delta introns}$
    b. Generation of $\alpha 1^{\Delta introns}$ and $\alpha 1^{WT\ compl.}$
    c. Generation of $\alpha 2^{+}$
    d. Generation of $\alpha 2^{++}$
    e. Generation of $\alpha 2^{+++}$
    f. Generation of $\alpha 1^{\Delta c\text{-}term}$
    g. qRT-PCR primers
    h. Genotyping primers

3. Appendix Text
    a. Mathematical model for microtubule growth from a fixed number of nucleation sites
    b. Estimation of model parameters for comparison between mathematical modelling results and experiments
    c. Appendix Figure S10.

4. Appendix References

Appendix Supplementary Figures 1- 9 and figure legends

Figure S1.

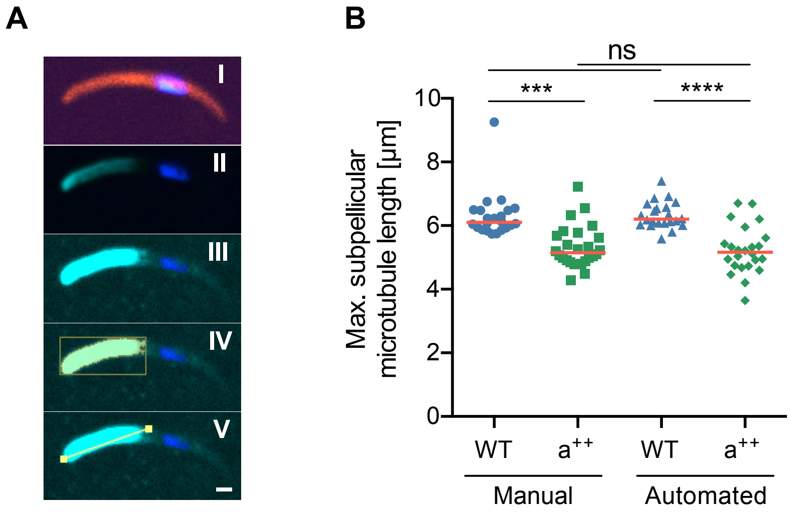

Principle workflow for quantitative analysis of microtubule length and intensity using Volocity Analysis 6.3 (Perkin Elmer)

A. A sporozoite is identified by the cytoplasmic mCherry signal (red) and the Hoechst staining (blue) of the nuclear DNA (I). The dynamic range of the original SiR-tubulin staining (cyan, II) was adjusted (III) to the weakest microtubule staining near the nucleus. The program quantifies original intensity levels of the SiR-tubulin stain (yellow, IV) and maximal microtubule length (V). Scale bar: 1 µm.

B. Comparison of manual and automated quantification of microtubule length from WT and $\alpha2^{++}$ sporozoites (see Fig EV10) labelled with SiR-tubulin reveals significant differences between the parasite lines but similar measurements between the two different quantification methods. *** and **** indicate p<0.001, and p<0.0001, respectively; ns: not significant; Kruskal-Wallis-test. Red line indicates median.

Figure S2.

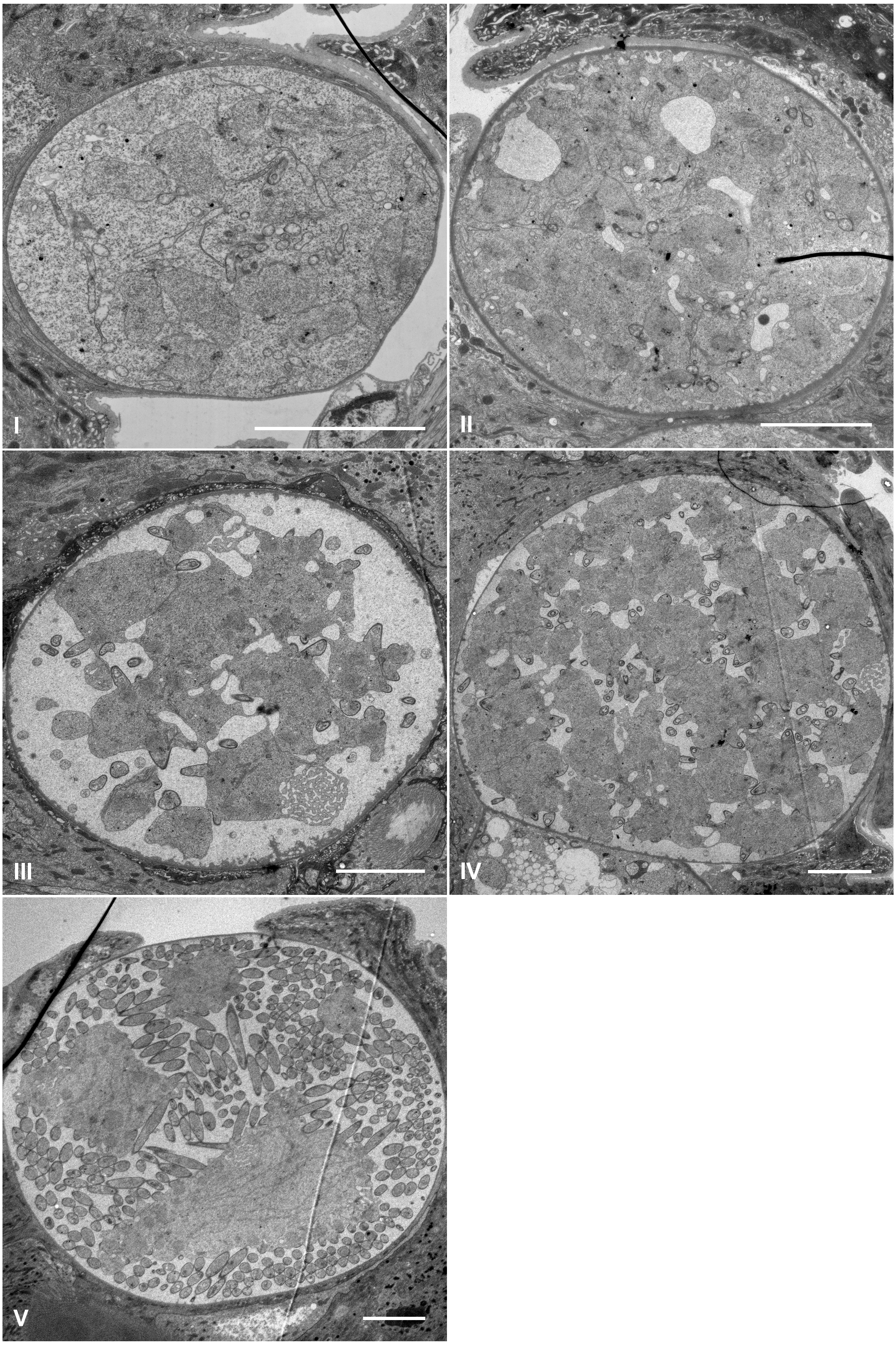

Transmission electron microscopy (TEM) of developing WT sporozoites

I-VI: Early to late stages of oocysts. Scale bars: 5 µm.

Figure S3.

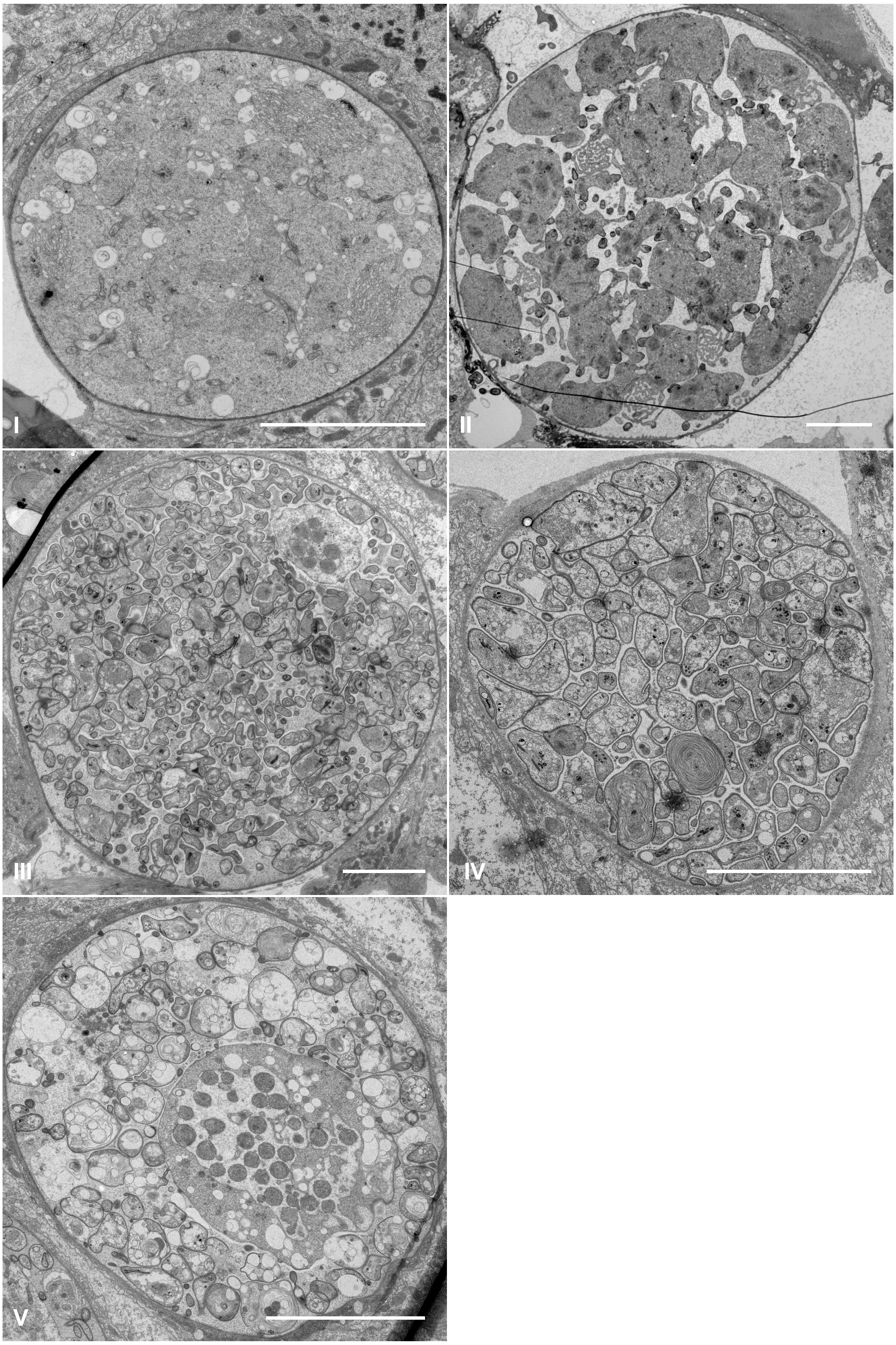

Transmission electron microscopy (TEM) of developing *α1-tubulin(-)* sporozoites

I-VI: Early to late stages of oocysts. Scale bars: 5 µm. Note the absence of slender sporozoite forms as in Fig EV4 panels V and VI.

Figure S4.

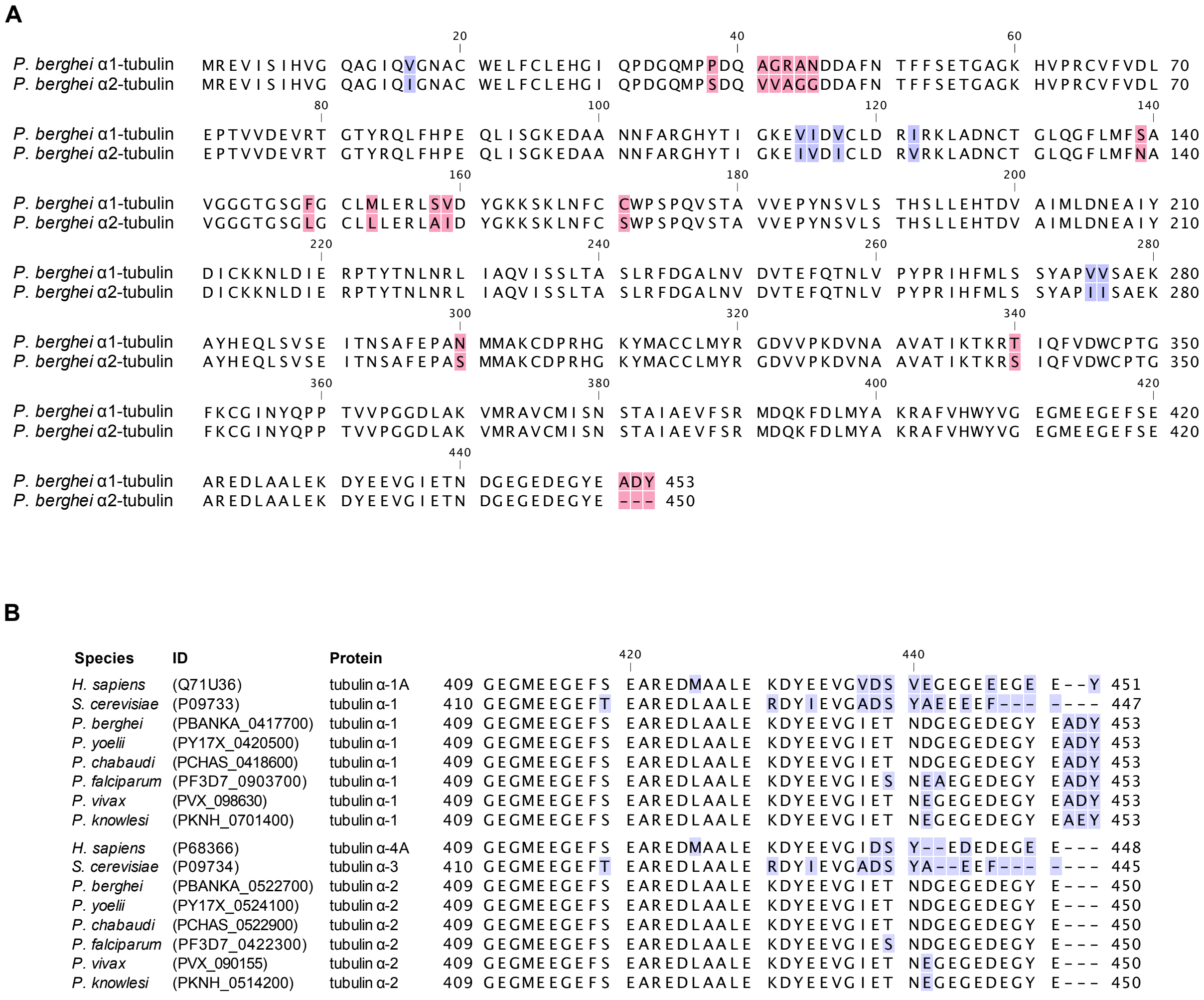

Differences between α1- and α2-tubulin in *Plasmodium berghei*

A. Alignment of α1- and α2-tubulin from *P. berghei* with the divergent amino acids that were changed in the chimeras highlighted in red. Isoleucines to valines were not changed in the α2^{+} chimera and are highlighted in blue (Aurrecoechea *et al*, 2009).

B. Alignment of the C-terminus of α1- and α2-tubulin from different *Plasmodium* species, human (*Homo sapiens*) and yeast (*Saccharomyces cerevisiae*). Sequences were retrieved from PlasmoDB (version 36) (Aurrecoechea *et al*, 2009) and UniProt (2018) (Bateman *et al*, 2017). Blue shading highlights differences to *P. berghei* α2-tubulin.

Figure S5.

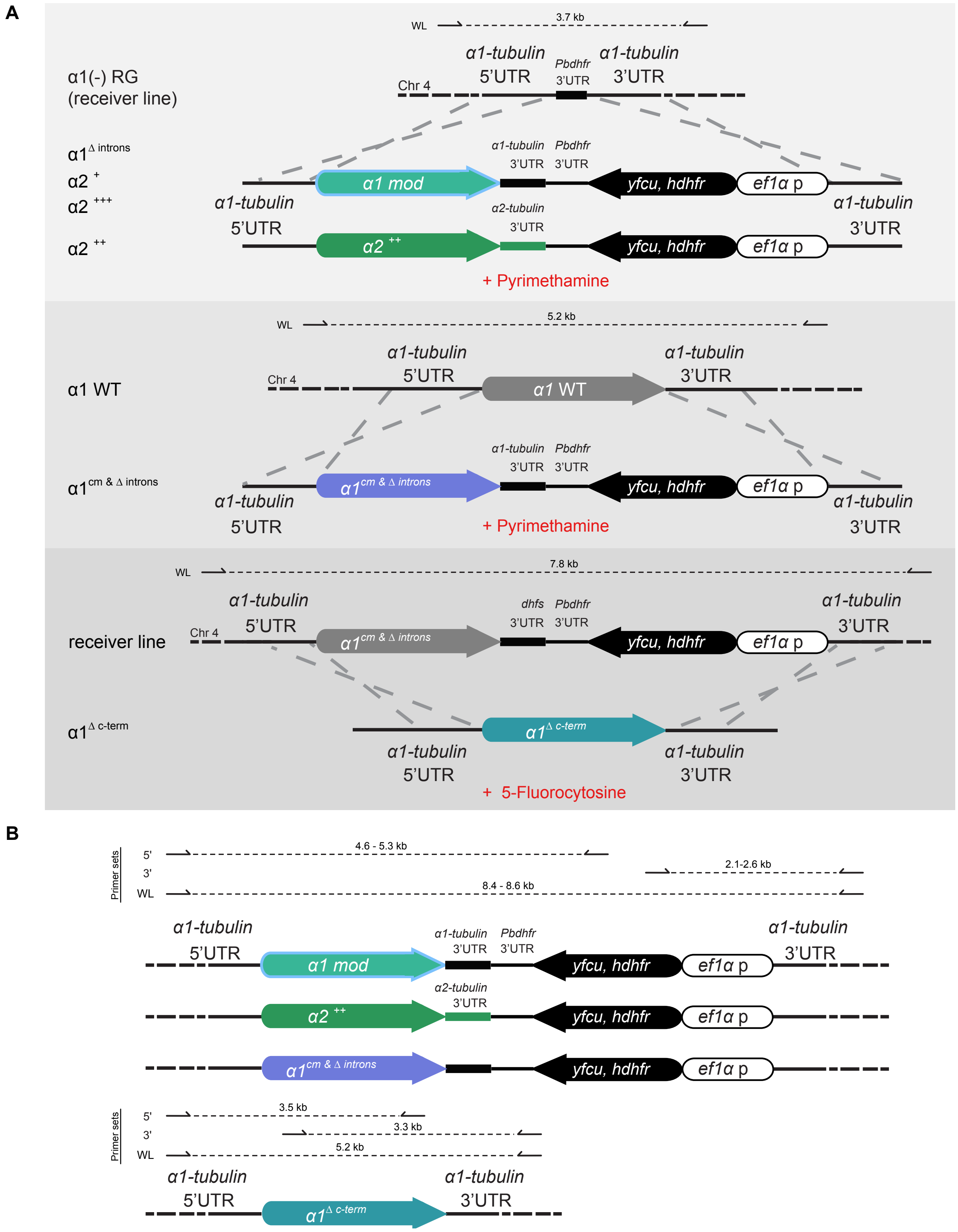
A
WL
3.7 kb
α1(-) RG
(receiver line)
Chr 4
α1-tubulin
5'UTR
Pbdhfr
3'UTR
α1-tubulin
3'UTR
α1Δ introns
α2 +
α2 +++
α2 ++
α1 mod
α1-tubulin
3'UTR
Pbdhfr
3'UTR
yfcu, hdhfr
ef1α p
α1-tubulin
5'UTR
α2-tubulin
3'UTR
α1-tubulin
3'UTR
α2 ++
+ Pyrimethamine
WL
5.2 kb
α1 WT
Chr 4
α1-tubulin
5'UTR
α1 WT
α1-tubulin
3'UTR
α1cm & Δ introns
α1cm & Δ introns
α1-tubulin
3'UTR
Pbdhfr
3'UTR
yfcu, hdhfr
ef1α p
α1-tubulin
5'UTR
α1-tubulin
3'UTR
+ Pyrimethamine
WL
7.8 kb
receiver line
Chr 4
α1-tubulin
5'UTR
α1cm & Δ introns
dhfs
3'UTR
Pbdhfr
3'UTR
yfcu, hdhfr
ef1α p
α1-tubulin
3'UTR
α1Δ c-term
α1Δ c-term
α1-tubulin
5'UTR
α1-tubulin
3'UTR
+ 5-Fluorocytosine
B
Primer sets
5'
3'
WL
4.6 - 5.3 kb
2.1-2.6 kb
8.4 - 8.6 kb
α1-tubulin
5'UTR
α1 mod
α1-tubulin
3'UTR
Pbdhfr
3'UTR
yfcu, hdhfr
ef1α p
α1-tubulin
3'UTR
α2 ++
α2-tubulin
3'UTR
α1cm & Δ introns
Primer sets
5'
3'
WL
3.5 kb
3.3 kb
5.2 kb
α1-tubulin
5'UTR
α1-tubulin
3'UTR
α1Δ c-term

C

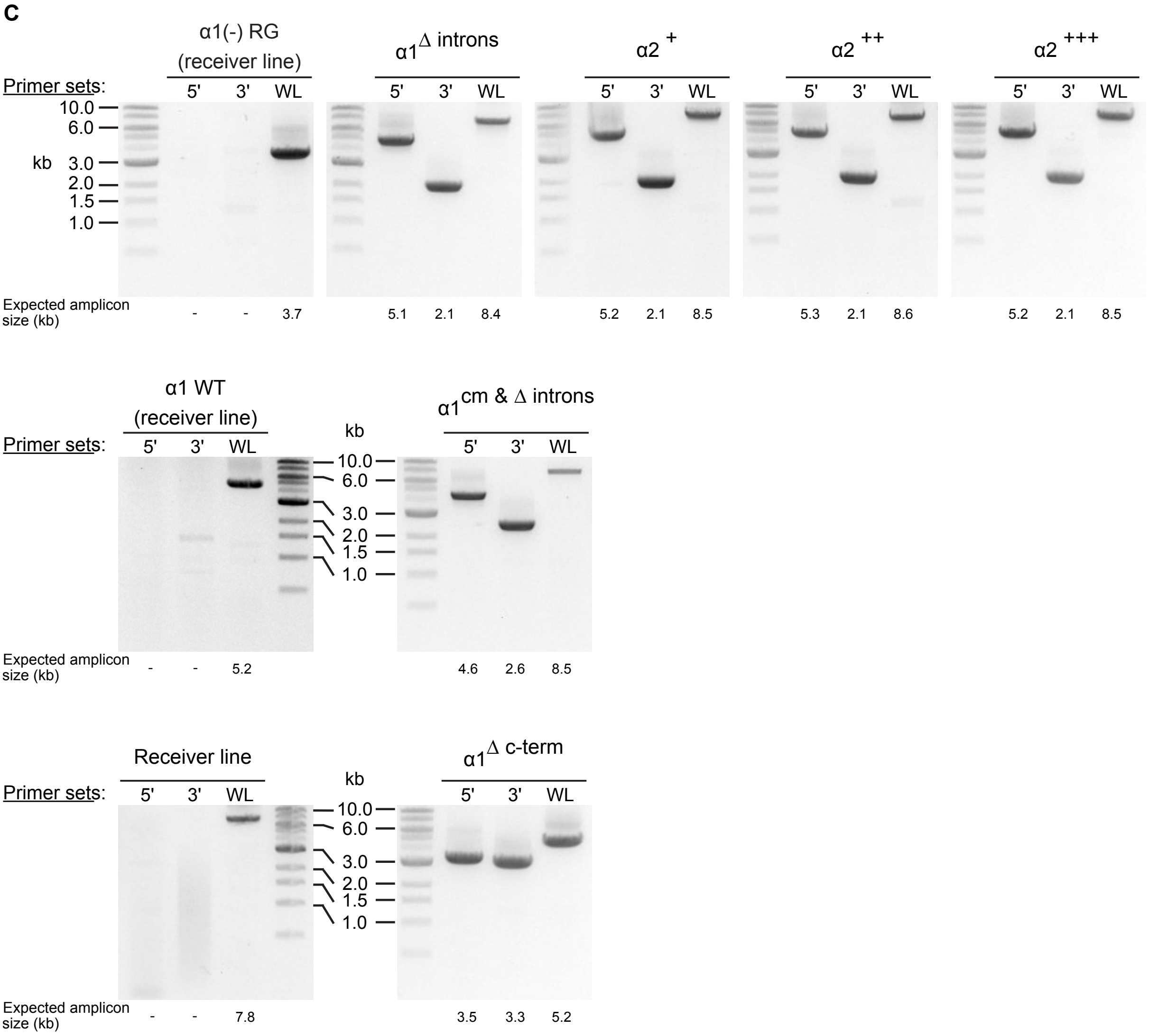

Generation of a series of parasite lines expressing different *α-tubulin* versions

A, B Cartoons showing the complementation and replacement strategies (**A**) used to generate the different transgenic parasite lines (**B**) expressing *α1-tubulin* variants or *α2-tubulin* in place of the endogenous *α1-tubulin*. Primers used for PCR analysis in (**C**) and amplicon sizes are indicated. Different strategies are indicated with different grey shading. Note that only the α1$^{\Delta c\text{-}term}$ parasite line could be generated with a gene-in-marker-out approach.

C. PCR analysis of the different parasite lines next to their receiver lines. Numbers indicate expected amplicon sizes. Please note that the first gel (α1-RG receiver line) is duplicated from Figure EV2B for comparison.

Figure S6.

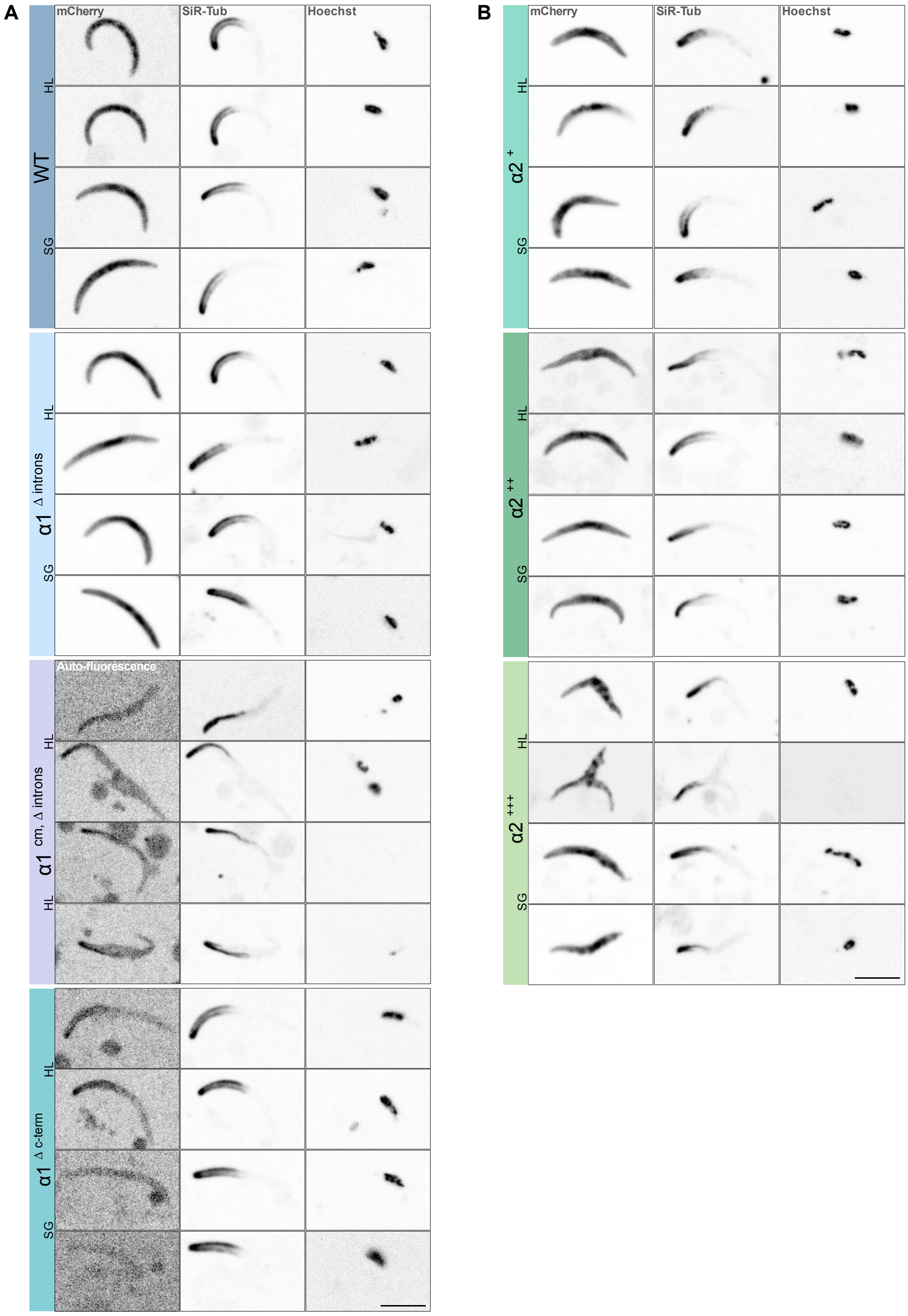

Spinning disc confocal microscopy sporozoites

Images of hemolymph (HL) and salivary gland (SG) derived sporozoites from the different parasite lines to highlight differences in shape, microtubule length and presence of nuclei.

A, B Sporozoites from parasite lines expressing *α1-tubulin* (**A**) or *tubulin* chimeras (**B**). Scale bars: 5 µm. Note that for non-fluorescent parasite lines the auto-fluorescence was used to illustrate their shape. Also note the absence of nuclei in some parasites.

Figure S7.

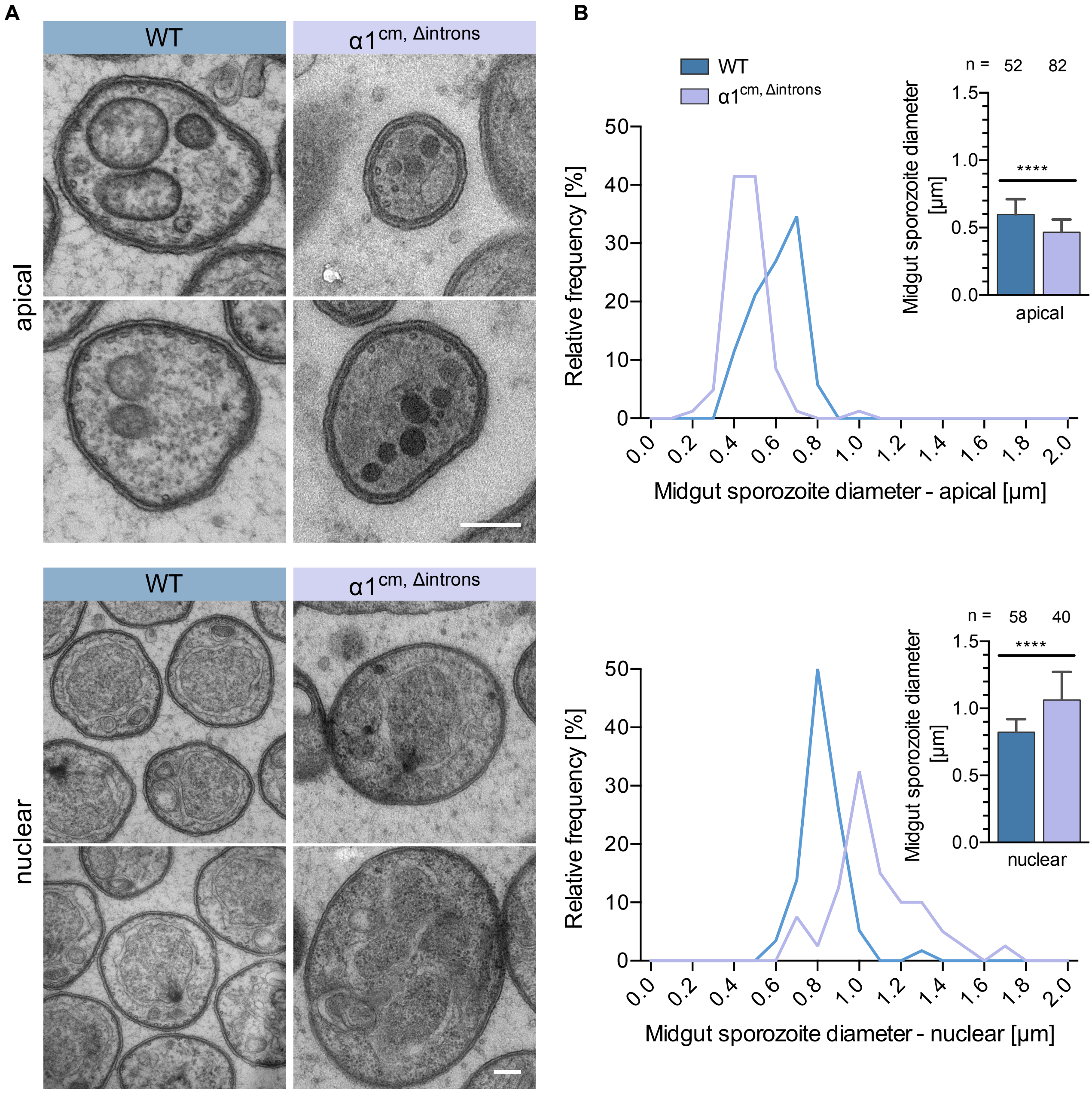

Different shape of sporozoites expressing α1$^{cm,\Delta introns}$ tubulin as determined by TEM

A. TEM images from WT and α1$^{cm,\Delta introns}$ tubulin expressing sporozoites from the apical (top) and nuclear (bottom) region. Note that the mutant shows smaller cross-sections at the apical end but larger cross-sections at the nucleus. Scale bars: 200 nm. Images are reproduced from Figure 4E and Figure 5E shown here again with a focus on the diameter.

B. Quantification of sporozoite diameters from the apical (top) and nuclear (bottom) region.

Figure S8.

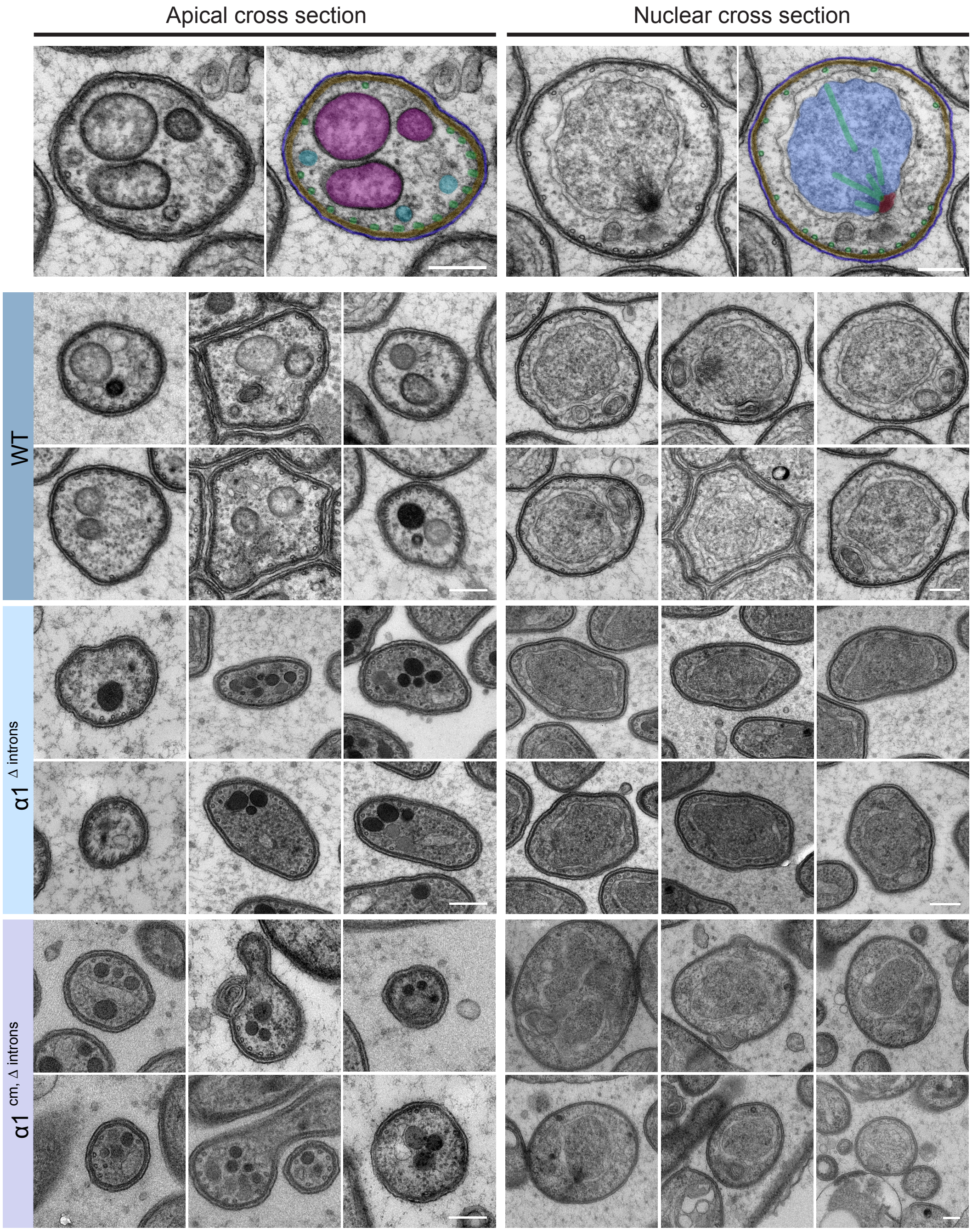

Apical and nuclear sporozoite cross sections of WT, $\alpha1^{\Delta introns}$ and $\alpha1^{cm\&\Delta introns}$ parasites

Gallery of TEM images from the apical and nuclear regions showing the different numbers of microtubules (green in overview example) in oocyst sporozoites from lines expressing *α1-tubulin*. Lilac: rhoptries; cyan: micronemes (apical section); blue: nucleus. Scale bars: 200 nm. Note that the colored example images are taken from Figures 4 and 5.

Figure S9.

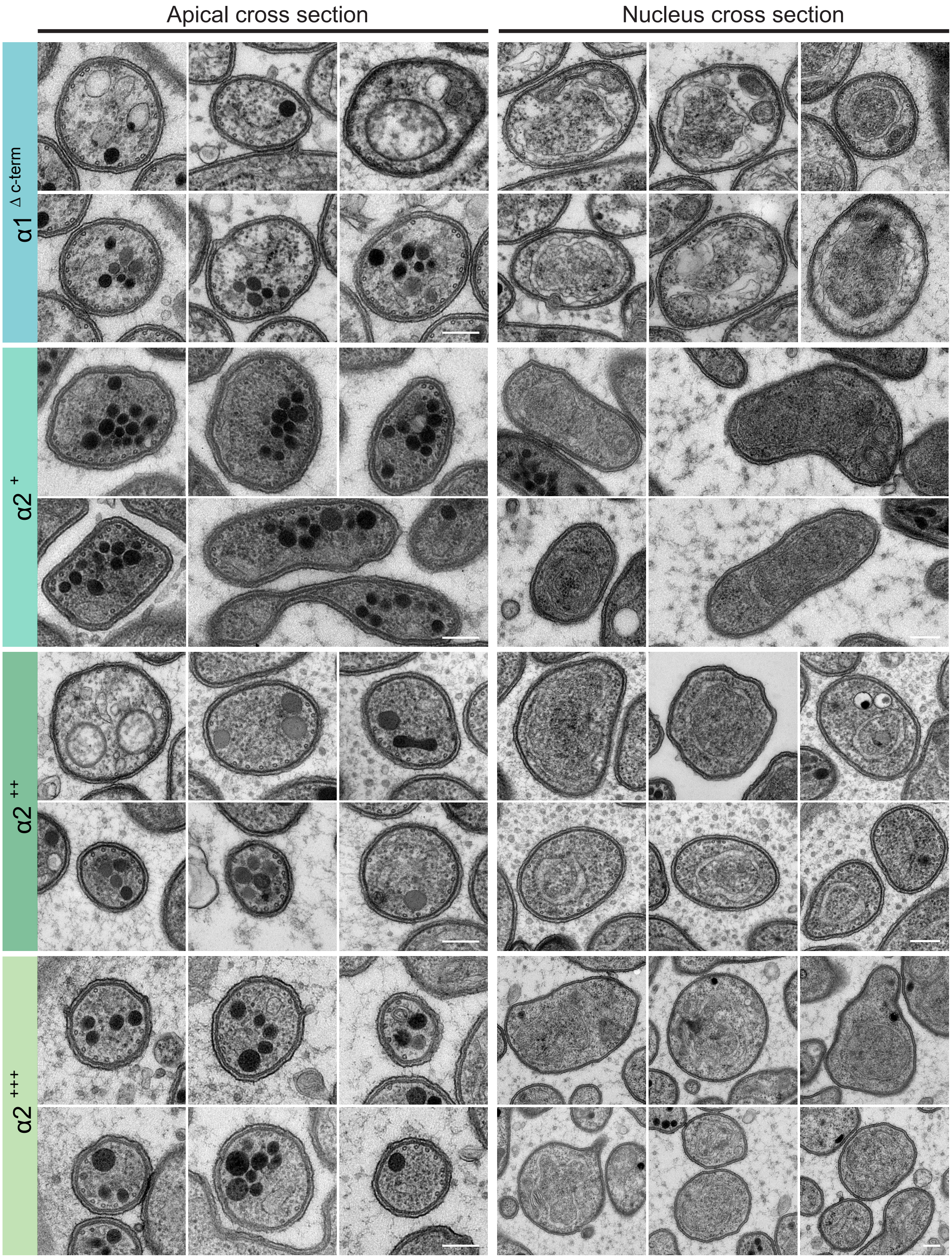

Apical and nuclear sporozoite cross sections of α1$^{\Delta c\text{-}term}$, α2^{+}, α2^{++}, α2^{+++} parasites
Gallery of TEM images from the apical and nuclear regions showing the different numbers of microtubules (green in overview example) in oocyst sporozoites from lines expressing *tubulin* chimeras.

Appendix Table S1.

Primers

Generation of α1$^{cm\&\Delta introns}$

Number	Primer sequence (5' to 3')
**766**	ACGCGTCGACGGATATTTAATGTTTTCAGTTTTCCACT
**767**	GGAATTCTTTTACTTGTATATTATAAAATAAACAATTGTTTTAAAATATAG
**768**	GGAATTCATGCGTGAGGTTATCTCTATCC
**769**	CCTTAATTAATTAGTAATCAGCTTCGTAACCC
**770**	CCTTAATTAAATTATTTCTTATCATTATAATGGTAAAAAAATTAAAAAG
**771**	GATATCCTTATATATATTTATACATTTCCTAAAATTATTAAACTAAT
**772**	CCCAAGCTTATTATTTTCTTATCATTATAATGGTAAAAAAATTAAAAAG
**773**	CCGCTCGAGGATAAAACAAAGACAAACTAAATAAATATAAGATAAAG
**838**	CGCCGGATCAAGCCGGCCGTG
**839**	CACGGCCGGCTTGATCCGGCG

Generation of α1$^{\Delta introns}$ and α1$^{WT\ compl.}$

Number	Primer sequence (5' to 3')
**1289**	CAATTGTTTATTTTATAATATACAAGTAAAAGCCTAGGATGAGAGAAGTAATAAGTATACATGTAG
**1290**	TAATTTTTTTACCATTATAATGATAAGAAAATAATGACGTCTTAATAGTCTGCCTCATATCC

Generation of α2^{+}

Number	Primer sequence (5' to 3')
**1305**	ACAATTGTTTATTTTATAATATACAAGTAAAAATGAGAGAAGTAATAAGTATACATG
**1306**	ACCAGGTTGTGGCAGGTGGTGATGATGCTTTTAATACCTTTTTTTCAGA
**1307**	CACCACCTGCCACAACCTGGTCACTAGGCATTTGACCATC
**1308**	GGATGTTTATTATTAGAAAGATTGGCTATTGATTATGGAAAAAAATCAAAGTTAAATTTTTGTTCATGGCCATCACC
**1309**	GCCAATCTTTCTAATAATAAACATCCAAGTCCACTTCCAGTACCACCTCCAACAGCATTAAACATCAAAAATCC
**1310**	CAGCATTTGAACCAGCATCTATGATGGCAAAATGTGATCC
**1311**	CACATTTTGCCATCATAGATGCTGGTTCAAATGCTGAG
**1312**	TATAAAAACGAAAAGATCTATTCAATTTGTTGATTGGTGC
**1313**	AATCAACAAATTGAATAGATCTTTTCGTTTTTATAGTGGC
**1625**	AATTTTTTTACCATTATAATGATAAGAAAATAATTTACTCATATCCTTCATCTTCTCC

Generation of α2^{++}

Number	Primer sequence (5' to 3')
**1283**	ACAATTGTTTATTTTATAATATACAAGTAAAAGAATTCATGAGAGAAGTTATTAGCATCC
**1468**	GTTTTTTCCTTCAATTTCGATGGGTACCCAAATCACATGATAAAATTATTATGC

Generation of α2^{+++}

Number	Primer sequence (5' to 3')
**767**	GGAATTCTTTTACTTGTATATTATAAAATAAACAATTGTTTTAAAATATAG
**1277**	GGATATGAATAAGACGTCATTATTTTCTTATCATTATAATGGTAAAAAAATT
**1278**	TGATGTTTTTTCCTTCAATTTCGATGGGTACCGATAAAACAAAGACAAACTAAATAAATATAAG
**1279**	GCTGGTAATTAAAGATATTATAATACTCTTGTATATCTTC
**1280**	AAGCTCCCTAAAGAAAAATTAATTAGAATTTTGTAAATAC
**1281**	AAACATGTAAGGAAATAAGAATTATATATATTTCAATAAT
**1282**	TCTAGGGACCTGTTAAAATTTGATCAAATATATATATGTG
**1283**	ACAATTGTTTATTTTATAATATACAAGTAAAAGAATTCATGAGAGAAGTTATTAGCATCC
**1284**	AGTATTATAATATCTTTAATTACCAGCAAGCATTTCCGAT
**1285**	ATTCTAATTAATTTTTCTTTAGGGAGCTTTTTTGCCTAGA
**1286**	TGAAATATATATAATTCTTATTTCCTTACATGTTTTCCAGCCCCAG
**1287**	TATTTGATCAAATTTTAACAGGTCCCTAGATGTGTATTCG
**1288**	AGAAAATAATGACGTCTTATTCATATCCTTCATCTTCTCC
**1599**	CGATCGGTCGACGTTTTATGTTTTCTTAATTGTTTCTGG
**1600**	GCTATGGGTACCATTGTCGTGGATTAGCAACG

Generation of α1$^{\Delta\text{c-term}}$

Number	Primer sequence (5' to 3')
**1305**	ACAATTGTTTATTTTATAATATACAAGTAAAAATGAGAGAAGTAATAAGTATACATG
**1315**	ATGATAAGAAAATAATTTACTCATATCCTTCATCTTCTCC

qRT-PCR primers

Number	Name	Primer sequence (5' to 3')
**1344**	*18S rRNA*	AAGCATTAAATAAAGCGAATACATCCTTAC
**1345**	*18S rRNA*	GGAGATTGGTTTTGACGTTTATGTG
**qP3**	*CSP*	GTTAAACAGATCAGGGATAGTATCACAGAGG

**qP4**	*CSP*	TTCAGTATCAATATCTTCTAAGGTCAAATCTTCTGC
**qP31**	*α1*	CCTCCTGACCAGGCTGGTAG
**qP32**	*α1*	GGGTGAAATAACTGGCGATATGTGC
**qP33**	*α2*	GCCCAGTGATCAAGTTGTGGC
**qP34**	*α2*	CAGGGTGAAATAATTGGCGATAGGTTC
**qP43**	*α1-cm*	TCAAGCCGGCCGTGCTAAC
**qP44**	*α1-cm*	GTTCCGGATGGAACAGTTGACG
**qP45**	*α2+*	AGCTATTTTGTTTAGAACATGGTATACAGCCC
**qP46**	*α2+*	GTTGGTTCTAAGTCAACAAAAACACAACGT

Genotyping primers

Number	Primer sequence (5' to 3')
**187**	TTCTACTGAAGAGGTTGTGGTC
**417**	GCTACATTCACACATACATGCG
**960**	TAATTCAAAGGGACGAGG
**1279**	GCTGGTAATTAAAGATATTATAATACTCTTGTATATCTTC
**1313**	AATCAACAAATTGAATAGATCTTTTCGTTTTTATAGTGGC
**1598**	GCAGGCATATGTAGAGCC

Appendix Text.

Mathematical model for microtubule growth from a fixed number of nucleation sites

To study how microtubule number and length depend on tubulin expression levels, we develop a mathematical model that describes how tubulin dimers bind to a fixed number of microtubule nucleation sites, form critical nuclei and then promote microtubule growth. In sporozoites, there are exactly $S_0 = 16$ nucleation sites and there is no evidence for any dynamical instability during microtubule growth. Previous modeling studies explaining in-vitro microtubule growth dynamics suggested that the critical nucleus contains around 12 dimers, corresponding roughly to one ring at the base of the microtubule (Kuchnir Fygenson *et al*, 1995; Flyvbjerg *et al*, 1996; Roostalu & Surrey, 2017). We found that the experimental data can be explained best if formation of the critical nucleus is divided into two steps. First p tubulin dimers bind to the fixed number of nucleation sites and then a critical nucleus forms at the bound sites after addition of n tubulin dimers, with $p + n = 12$. Once a critical nucleus is formed, the microtubule steadily grows by addition of tubulin dimers. Because sporozoites have a relatively small volume, we consider absolute numbers rather than concentrations. Based on our observations that sporozoite microtubules grow to a characteristic maximum length of 6 μm, we consider a phenomenological microtubule growth function that restricts growth above $m_0 = 9750$ dimers, which is the number of dimers in a 6 μm long microtubule that contains 13 protofilaments of 8 nm sized dimers. With these assumptions, we arrive at the following set of equations:

$$\frac{dS_b}{dt} = k_b c^p \left(S_0 - S_b - N_{nuc}\right) - k_n c^n S_b \quad (1)$$

$$\frac{dN_{nuc}}{dt} = k_n c^n S_b \quad (2)$$

$$\frac{dM}{dt} = k_m c \left(N_{nuc} - \frac{M}{m_0}\right) \quad (3)$$

$$c = \left(c_0 - pS_b - nN_{nuc} - M\right) \quad (4)$$

Eq. (1) describes the binding of p tubulin dimers (with overall number c) to free nucleation sites $(S_0 - S_b - N_{nuc})$ with rate k_b. Eq. (2) states that a bound nucleation site S_b can form a critical nucleus upon addition of n dimers with rate k_n. Eq. (3) describes the growth of microtubule mass M, i.e., the total number of dimers in microtubule form. For a single nucleus ($N_{nuc} = 1$), this equation reduces to growth equation for a single microtubule which polymerizes at rate k_m until m_0 is reached. For growth of multiple microtubules, there is competition between dimers going into nucleation or growth. To establish a well-defined final microtubule length, the growth rate becomes negative when microtubule mass $M > N_{nuc}\, m_0$. Eq. (4) accounts for the decrease in available tubulin concentration due to their binding to nucleation sites and the formation of nuclei and microtubules. Here c_0 is the initial tubulin dimer number. Because we deal with a small system, we resolve discrete binding events. We use Gillespie algorithm to perform simulations of the stochastic version of the system of equations (1-4).

Estimation of model parameters for comparison between mathematical modelling results and experiments

The unknown model parameters in our simulations are k_b, k_n, k_m, p. In principle, the exact microtubule growth velocity k_m does not matter for our model, because it determines the time scale, which experimentally we cannot observe. To be specific, however, here we assume that the typical growth velocity is ~40 nm/s as measured for microtubule growth in vitro and in vivo (summarized in Table 1 of (Zelinski *et al*, 2012)). With 13 protofilaments and a dimer size of 8 nm, for a single-protofilament model as used here, the used growth velocity translates into a dimer association rate $k_m = \frac{40\text{x}13}{8} = 66\ \text{sec}^{-1}$ per dimer. Next, we compute the mean squared error (MSE) between simulation results with the experimental data for microtubule number, N_{nuc} and average microtubule length, $L\,(= M/N_{nuc})$. Mathematically, we define

MSE in MT number $= \sqrt{\sum_{c_0} \left(N_{nuc}^{sim} - N_{nuc}^{exp}\right)^2}$ and MSE in MT length $= \sqrt{\sum_{c_0}(L^{sim} - L^{exp})^2}$. We convert the experimentally measured tubulin expression level in units of dimer number by assuming that 43.7% (α1c-term mutant tubulin expression) of WT expression equals a dimer number $c_0 = 16(m_0 + p + n)$ and can form 16 full-length MTs. Here $(m_0 + p + n)$ is the minimum dimer number required to form a single microtubule of length m_0, where $p + n = 12$ is the number of dimers required to form a nucleus. The relation between dimer number and expression level indicates that WT contains more than double the dimer number required to form 16 microtubules. Next, we calculate total MSE by adding the MSE at different initial dimer number (for different strains) calculated from 300 independent stochastic simulations. Then we find the conditions that minimize MSE in MT number by varying three parameters, k_n, k_b *and* p. We observe that decreasing k_n to very low values increases the error in MT number (data not shown). Further, the error remains constant for large k_n values (data not shown). Therefore, we keep k_n fixed at a relatively large value ($10^{-10}\, sec^{-1}$ *per dimer*). Next, we vary p and find that the increasing p increases the minimum error in MT number but decreases the error in MT length (see Main Text). We also plot the average microtubule length when the 16th MT nucleus forms at WT dimer number in Figure S10. The plot shows that average MT length is low at high p values. Our experiments imply that all microtubules grow together, which suggests that microtubule growth is minimal before all nuclei are formed. Therefore, based on biologically relevant considerations, we select the optimal parameters ($k_b = 10^{-11}, k_n = 10^{-10}, k_p = 6\, sec^{-1}$ *per dimer*).

For the optimal parameters, the rate $\mathrm{k_m}$ is higher than $\mathrm{k_b}$ and $\mathrm{k_n}$ at all concentrations. The rate of binding $\mathrm{k_b c^p}$ and the rate of microtubule growth $\mathrm{k_m c}$ play the major roles in determining microtubule numbers and length. The rate of forming full-length microtubules is $\sim\mathrm{k_m c/m_0}$, which is the inverse of the time required to add $\mathrm{m_0}$ dimers to a nucleus. The rate of microtubule

formation is lower than the rate of binding (or nucleation) for concentration $c \geq 2.7\ m_0$. Figure S10 shows that the binding rate decreases and becomes comparable to the rate of microtubule formation as the initial concentration decreases. This leads to a significant decrease in concentration between nucleation events at low concentrations, which slows down further nucleation and thereby limits the number of nuclei at a given concentration.

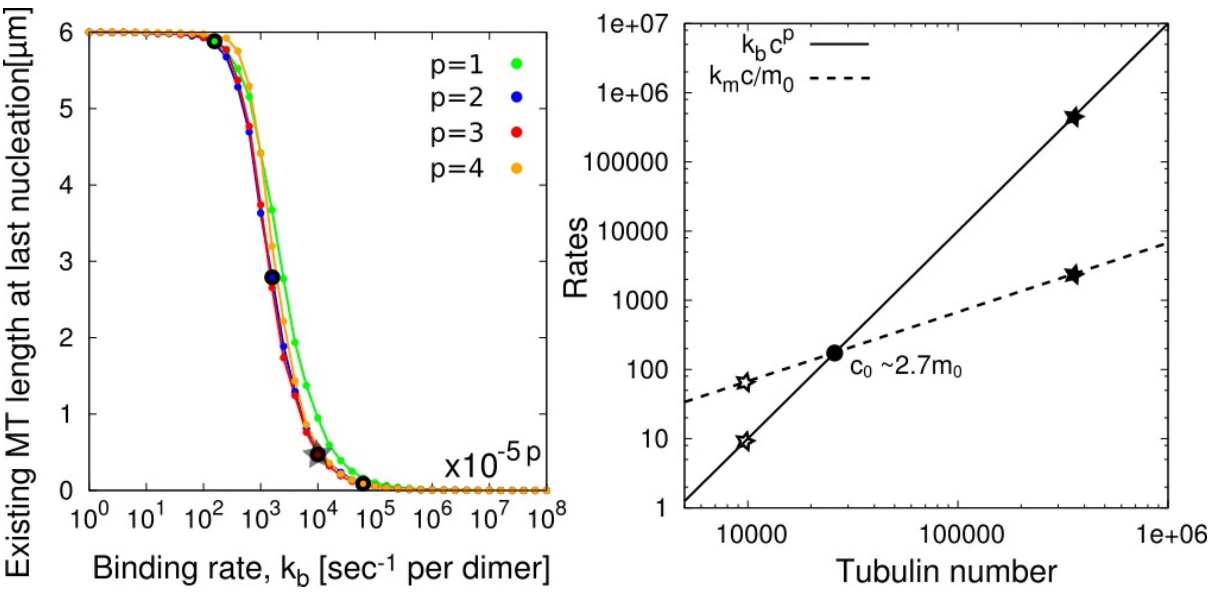

Appendix Figure S10.

A. The average length of existing microtubules (for WT) when the 16th MT nucleus forms decreases as the binding rate to nucleating sites is increased. The increase in binding cooperativity has an insignificant effect on the average length, however, the average length corresponding to MSE in MT number minima (open black circles) decreases considerably. The star symbols the optimal parameter chosen to reproduce experimental MT number vs concentration trend.

B. Tubulin dimer number determines the difference between the rate of microtubule formation (dashed curve) and the rate of binding to the nucleation sites (solid curve). For the chosen parameters, the binding rate to nucleation sites is faster than the rate of microtubule formation for the tubulin concentrations on the right side ($c > 2.7m_0$) of the curve intersection point

(black circle). On the left side ($c < 2.7m_0$), microtubules form faster than nucleation occurs. The filled stars show WT tubulin dimer number and open stars show dimer number for forming a single microtubule.

Appendix References

Flyvbjerg H, Jobs E & Leibler S (1996) Kinetics of self-assembling microtubules: An 'inverse problem' in biochemistry. *Proc. Natl. Acad. Sci.* **93:** 5975–5979

Kuchnir Fygenson D, Flyvbjerg H, Sneppen K, Libchaber A & Leibler S (1995) Spontaneous nucleation of microtubules. *Phys. Rev. E* **51:** 5058–5063

Roostalu J & Surrey T (2017) Microtubule nucleation: Beyond the template. *Nat. Rev. Mol. Cell Biol.* **18:** 702–710

Zelinski B, Müller N & Kierfeld J (2012) Dynamics and length distribution of microtubules under force and confinement. *Phys. Rev. E* **86:** 1–15
