# Supplementary material for: Microtubule number and length determine cellular shape and function in Plasmodium
Source: EMBO J. 2019 May 24;38(15):e100984. doi: 10.15252/embj.2018100984 (PMC6669926; doi:10.15252/embj.2018100984)
Supplement: Supplementary file 2 — Expanded View Figures PDF [file EMBJ-38-e100984-s002.pdf]

Expanded View Figures

Figure EV1. Generation of *α1-tubulin*(-) parasite lines.

A *Plasmodium* genomes, like those of yeast, contain two *α1-tubulins* and one *β-tubulin*, while other protozoan such as *Toxoplasma gondii* or metazoans can contain many more. Data collected from (Ludueña & Banerjee, 2008) and PlasmoDB (version 36; Aurrecoechea *et al*, 2009).

B Genome localization and gene structure of *P. berghei* and *P. falciparum tubulin* genes. Note that *α1-tubulin* has a longer C-terminus than *α2-tubulin*.

C Strategy to delete *α1-tubulin* from the genome of *P. berghei*, strain ANKA (wild type) and from the double fluorescent *P. berghei* strain ANKA line "RG", which expresses two fluorescent proteins. Primers used for analysis in (D) and resultant amplicons (with length) are indicated. Pyrimethamine is used for positive selection, and 5-fluorocytosine is used for negative selection. Note the *Pbdhfr* 3′UTRs used for negative selection (green). WL: whole locus.

D PCR analyses showing the successful deletion of *α1-tubulin*. Numbers below lanes show expected amplicon sizes as indicated in (C). WL: whole locus.

E Blood-stage growth rate after injection of 100 WT control and *α1-tubulin*(-) parasites in 4 and 8 C57BL/6 mice, respectively. Note that a similar growth rate was seen in the PlasmoGEM screen for *α1-tubulin*(-) parasites (Bushell *et al*, 2017). One-way ANOVA test was used for statistical analysis.

F Numbers of oocysts per midgut from infected *Anopheles stephensi* mosquitoes from WT-like control (see Fig EV2) and *α1-tubulin*(-) parasite lines. *n* indicates numbers of investigated midguts.

Source data are available online for this figure.

© 2019 The Authors

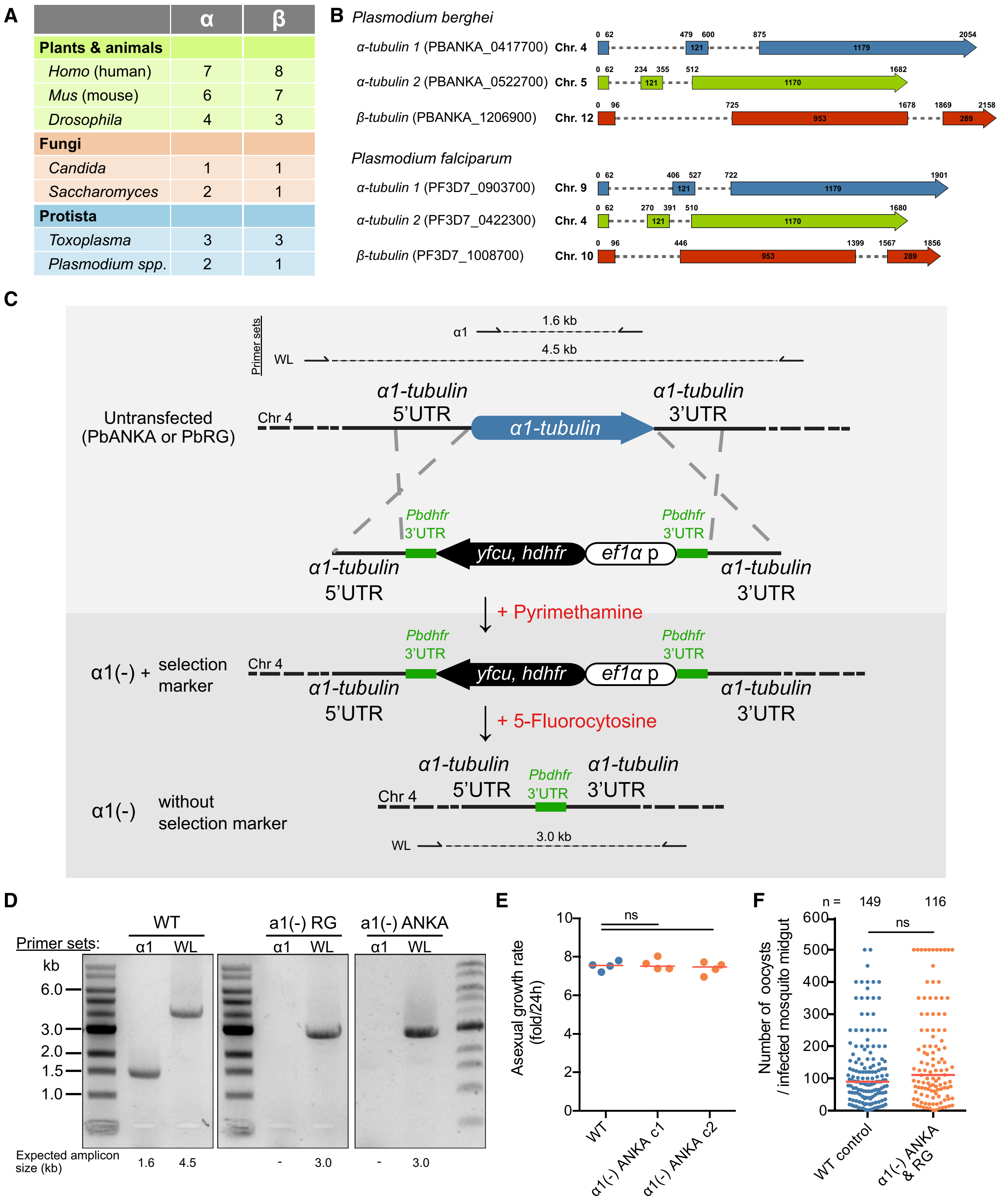

Figure EV1.

© 2019 The Authors

A

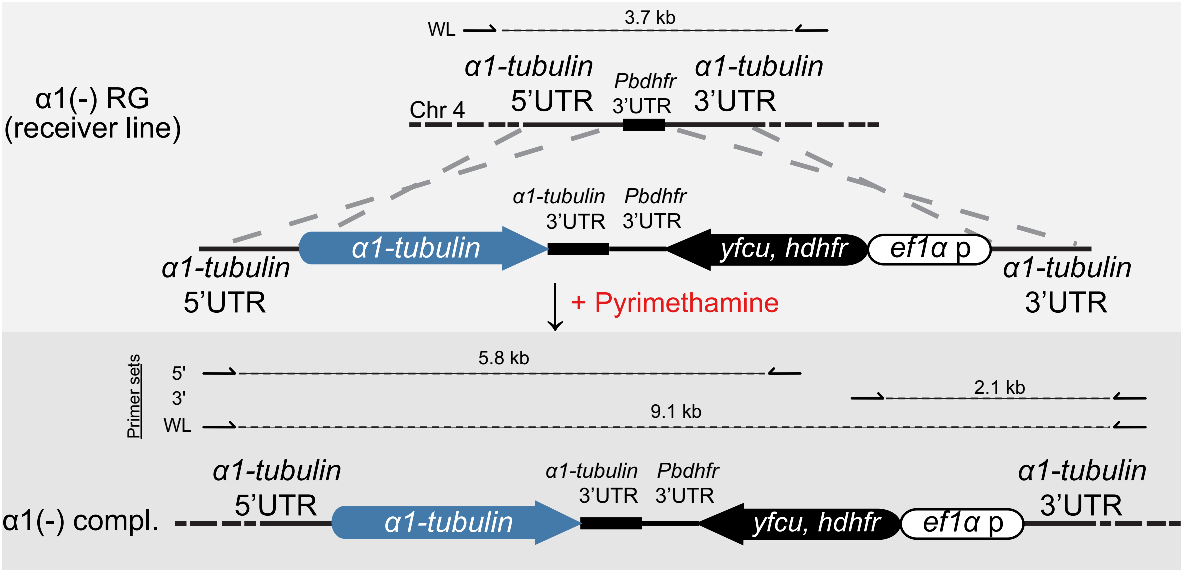

B

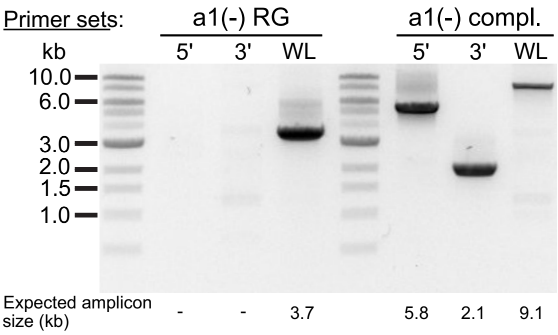

C

	Parasite line	WT ANKA	WT ANKA (RG)	α1(-)	α1(-) compl.
Midgut sporozoites	Sporozoites / mosquito (n)	93,000 (42)	97,800 (39)	0 (70)	88,000 (64)
	N° of apical subpellicular microtubules (n)	16 (lit.)	16 (11)	0 (35)	16 (23)
	Sporozoite morphology	+++	+++	n.a.	+++
Salivary gland sporozoites	Sporozoites / mosquito (n)	19,100 (38)	20,400 (34)	0 (67)	20,600 (61)
	Salivary gland invasion ratio	0.21	0.21	n.a.	0.23
	Infected mice by 10 mosquito bites / total mice (prepatency)	n.d.	8/8 (4.3)	n.a.	7/8 (3.9)
	Infected mice by 10.000 sporozoites injected i.v. / total mice (prepatency)	n.d.	8/8 (3.9)	n.a.	11/12 (3.7)

Figure EV2. Complementation of *α1-tubulin*(-) parasites with *α1-tubulin*.

A Cartoon showing the complementation strategy. Primers used for PCR analysis in (B) and amplicon sizes are indicated.

B PCR analysis of the complemented parasite line. Numbers indicate expected amplicon sizes.

C Comparative analysis of the complemented and *α1-tubulin*(-) parasite lines with two wild-type parasite lines (*P. berghei* strain ANKA and a *P. berghei* strain ANKA derived line expressing two fluorescent proteins and named RG). Sporozoite numbers from midgut and salivary glands of the different lines and the infectivity of salivary gland sporozoites for NIMR mice were analysed. Note that the 16 microtubules of the WT line are taken from the literature (lit.). Red letters indicate difference from the WT.

© 2019 The Authors

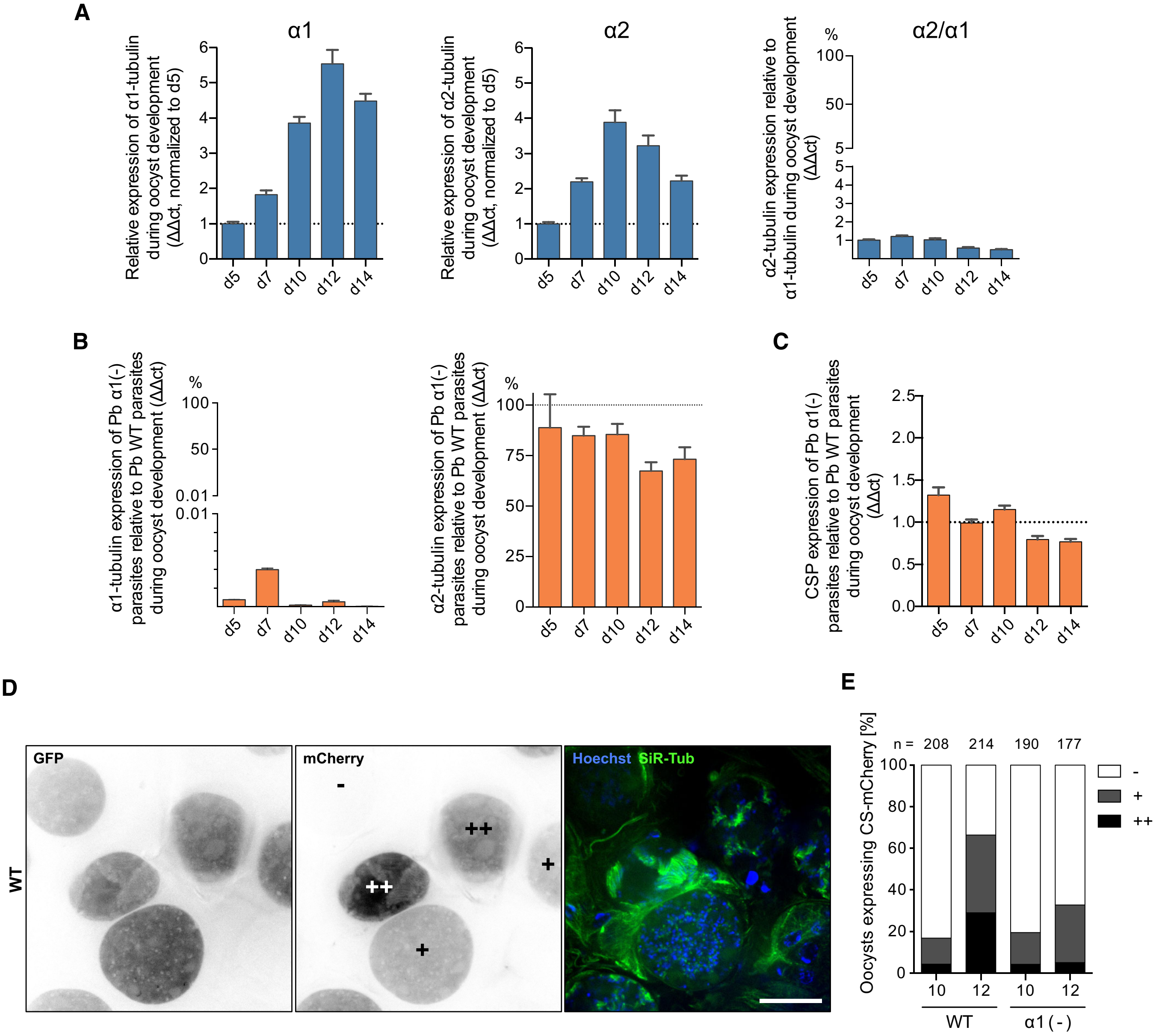

Figure EV3. Absence of microtubules delays CSP expression.

A, B mRNA levels as determined by qRT–PCR analysis of infected midguts at different times post-mosquito infection. Note that *α2-tubulin* is much less expressed in oocysts than *α1-tubulin* (A) and that *α2-tubulin* expression is unchanged in *α1-tubulin*(-) oocysts (B). Error bars indicate the standard deviation from the mean of technical duplicates.

C *CSP* expression of *α1-tubulin*(-) parasites relative to WT on different days post-mosquito blood meal. Relative expression was calculated via the ΔΔct method with error bars indicating the standard deviation of the mean calculated from technical duplicates.

D Live images of oocysts expressing GFP from the *ef1α* promoter and *mCherry* from the *csp* promoter. Microtubules were labelled with SiR-tubulin, and DNA was labelled with Hoechst in the merged image. Note that *CSP* expression is not seen in all oocysts (—) and that oocysts with stronger (+) and very strong (++) *CSP* expression progressed further in development as evidenced by strong SiR-tubulin staining and individualized nuclei. Scale bar: 10 μm.

E Quantification of oocyst maturation as judged from expression of CSP on days 10 and 12 post-blood meal. The number of total investigated oocysts is indicated above each column, and —, +, ++ indicate no, weak and strong *CSP* expression as indicated in panel D.

Source data are available online for this figure.

 © 2019 The Authors

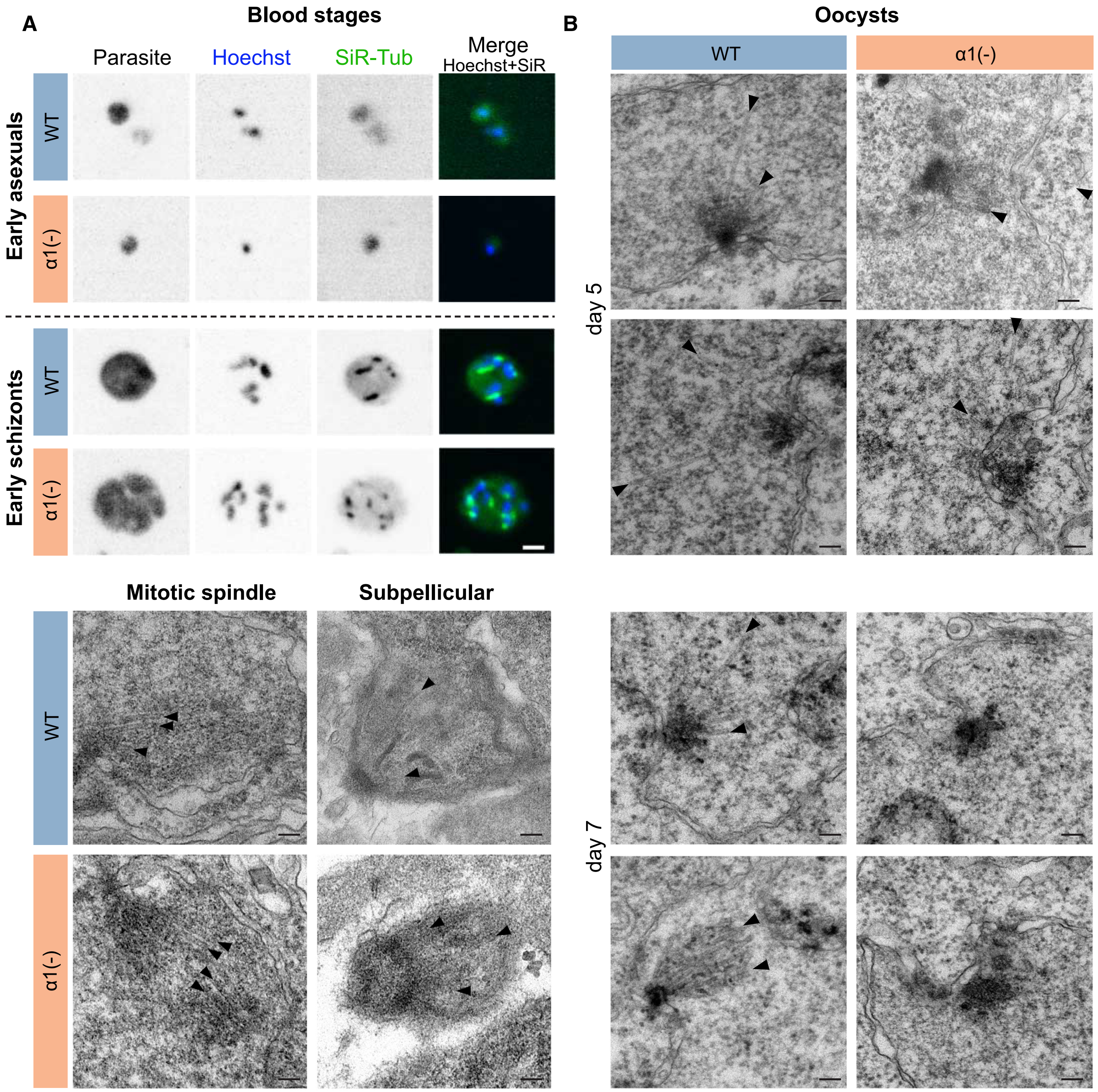

Figure EV4. Spindle formation as revealed by SiR-tubulin staining in blood stages and electron microscopy in oocysts.

A Microtubules in blood stages of wild-type and *α1-tubulin*(-) parasites. Top: SiR-tubulin staining of early, non-replicating ring stages (early asexuals) reveals a cytoplasmic background of the dye, while (hemi-) spindles are readily visualized in replicating schizonts by spinning disc confocal microscopy. Hoechst was used to label nuclei and a cytoplasmic fluorescent protein to highlight the parasite. Scale bars: 2 μm. Bottom: Transmission electron micrographs of wild-type and mutant showing microtubules (arrowheads) of mitotic spindles (left) and subpellicular microtubules (right). Scale bars: 100 nm.

B Transmission electron micrographs from oocysts at the indicated days post-mosquito infection from wild-type and *α1-tubulin*(-) parasites. While some microtubules (black arrowheads) can still be seen at day 5 (top 4 images) in the *α1-tubulin*(-) parasites, none are detectable from day 7 onwards (bottom 4 images). Scale bars: 100 nm.

© 2019 The Authors

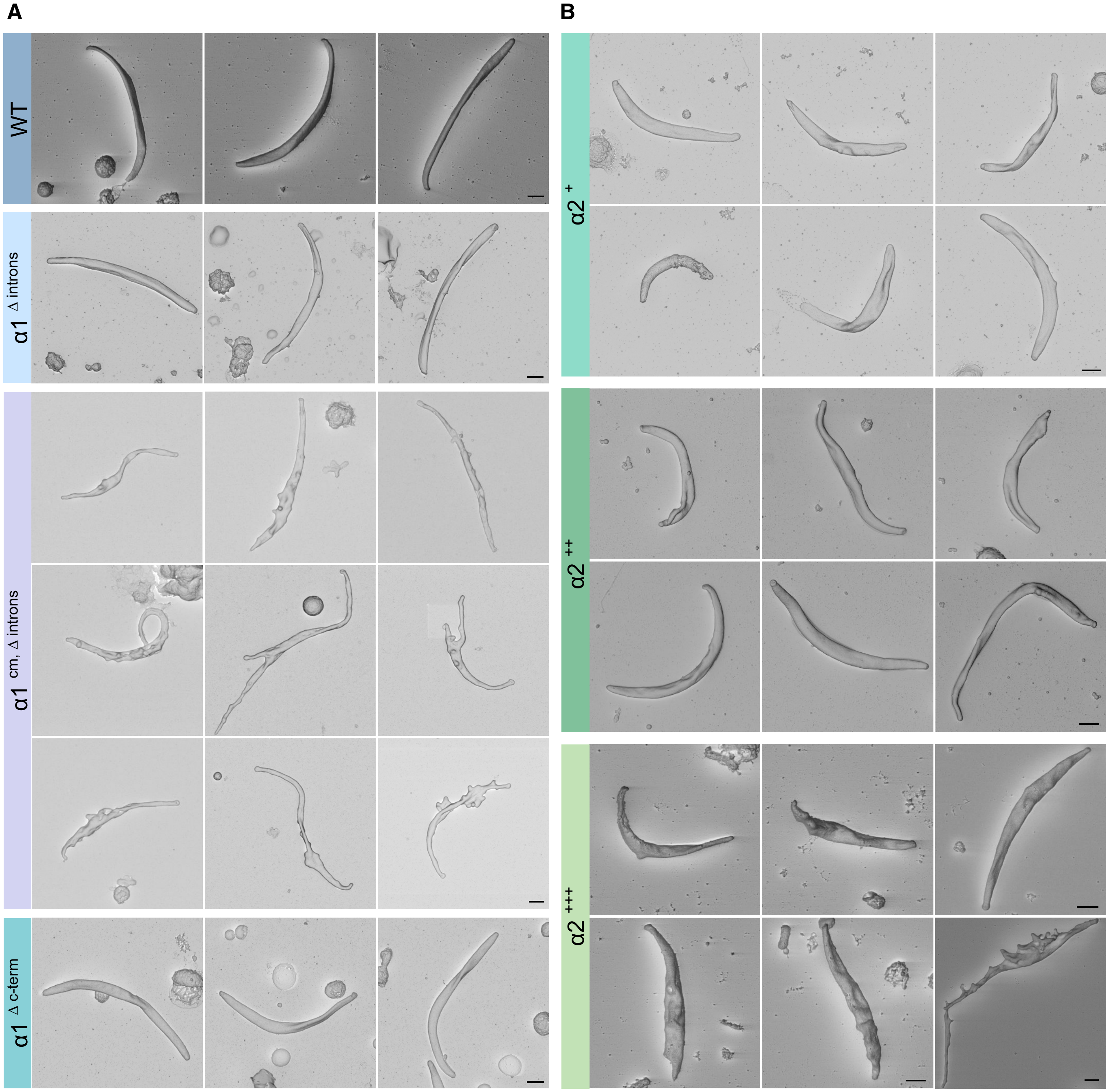

Figure EV5. Normal and aberrant sporozoite morphology in the generated parasite lines.

A, B Scanning electron micrographs of sporozoites from the indicated parasite lines expressing α1-tubulin (A) and tubulin chimeras (B). Scale bars: 1 μm.

© 2019 The Authors
