# Supplementary material for: Microtubule number and length determine cellular shape and function in Plasmodium
Source: EMBO J. 2019 May 24;38(15):e100984. doi: 10.15252/embj.2018100984 (PMC6669926; doi:10.15252/embj.2018100984)
Supplement: Supplementary file 3 — Movie EV1 [file EMBJ-38-e100984-s003.zip › 100984_MovieEV1.docx]

**Movie EV1 - Tomographic models of budding sporozoites**

3D rendered tomographic reconstructions of budding sporozoites from serial sections of wild type (left) and *α1-tubulin(-)* (right). Colors indicate: plasma membrane (blue), rhoptries (magenta), rootlet fiber (red), micronemes and small vesicles (yellow and brown), microtubules (green) and the nucleus (cyan). Please note that due to the used transparencies colors change during rotation. Scale bar: 1 µm.
