# Supplementary figures and images for: Microtubule number and length determine cellular shape and function in Plasmodium
Source: EMBO J. 2019 May 24;38(15):e100984. doi: 10.15252/embj.2018100984 (PMC6669926; doi:10.15252/embj.2018100984)

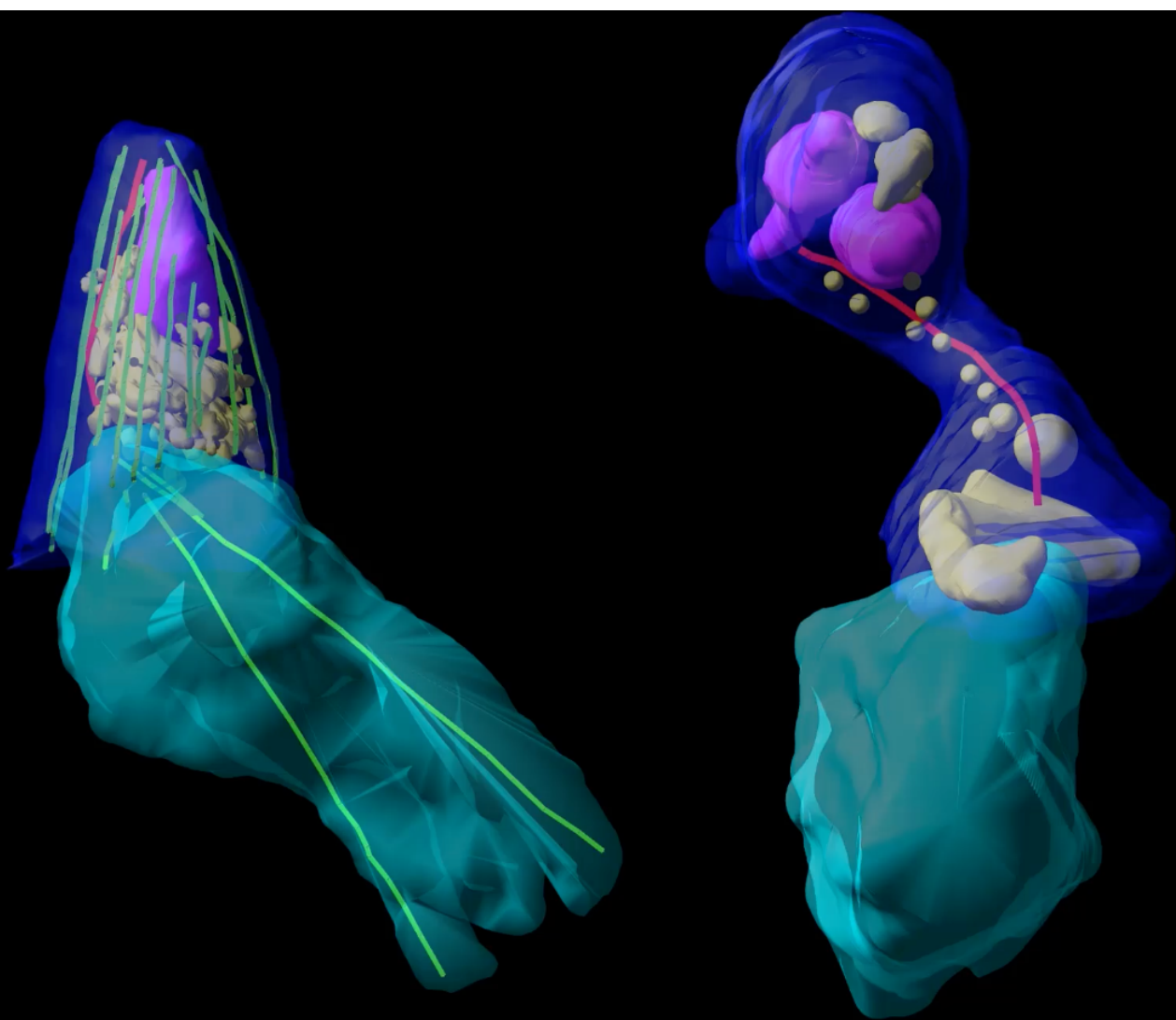

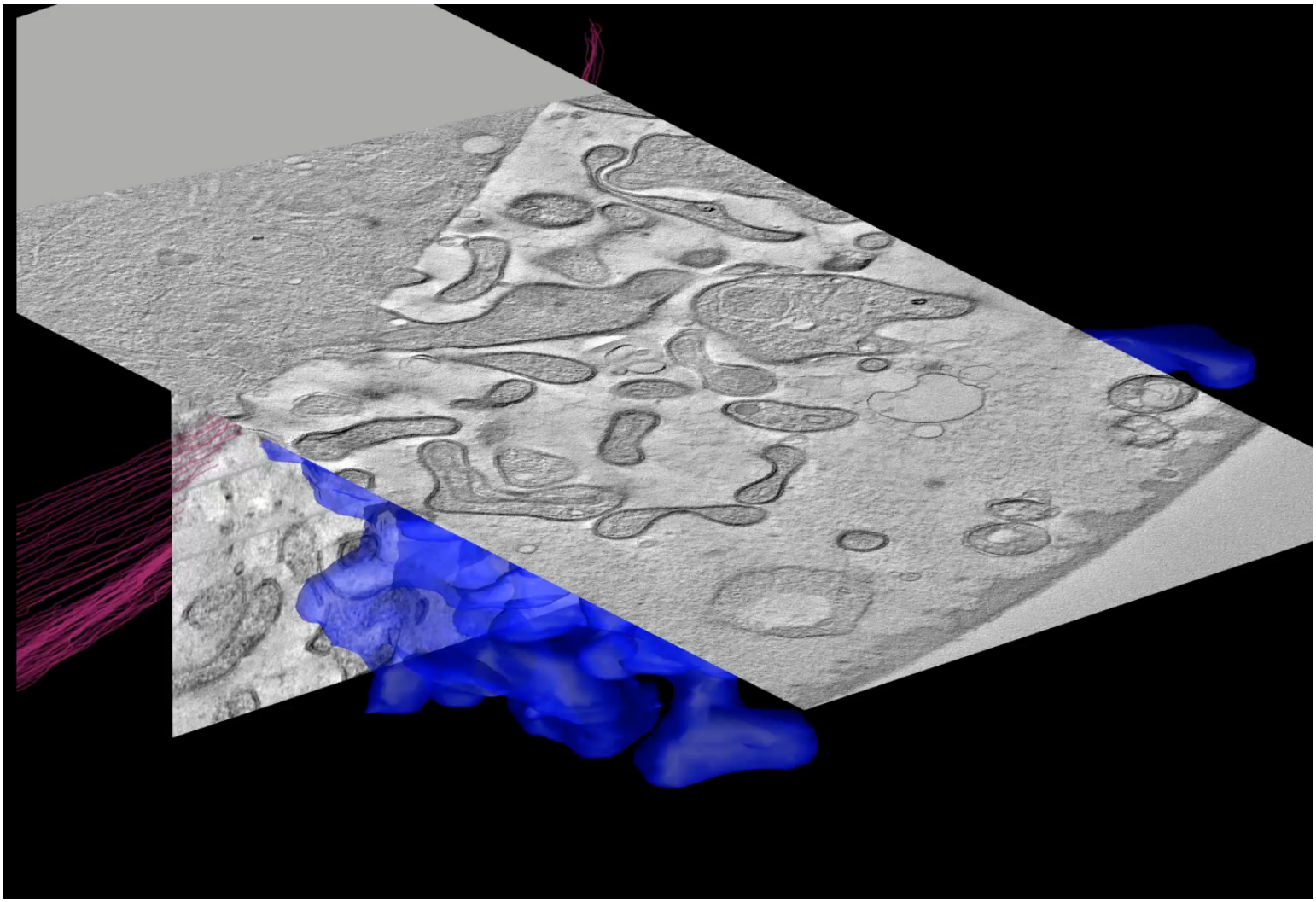

Supplement: Supplementary file 3 — Movie EV1 [file EMBJ-38-e100984-s003.zip › 100984_MovieEV1.pdf]

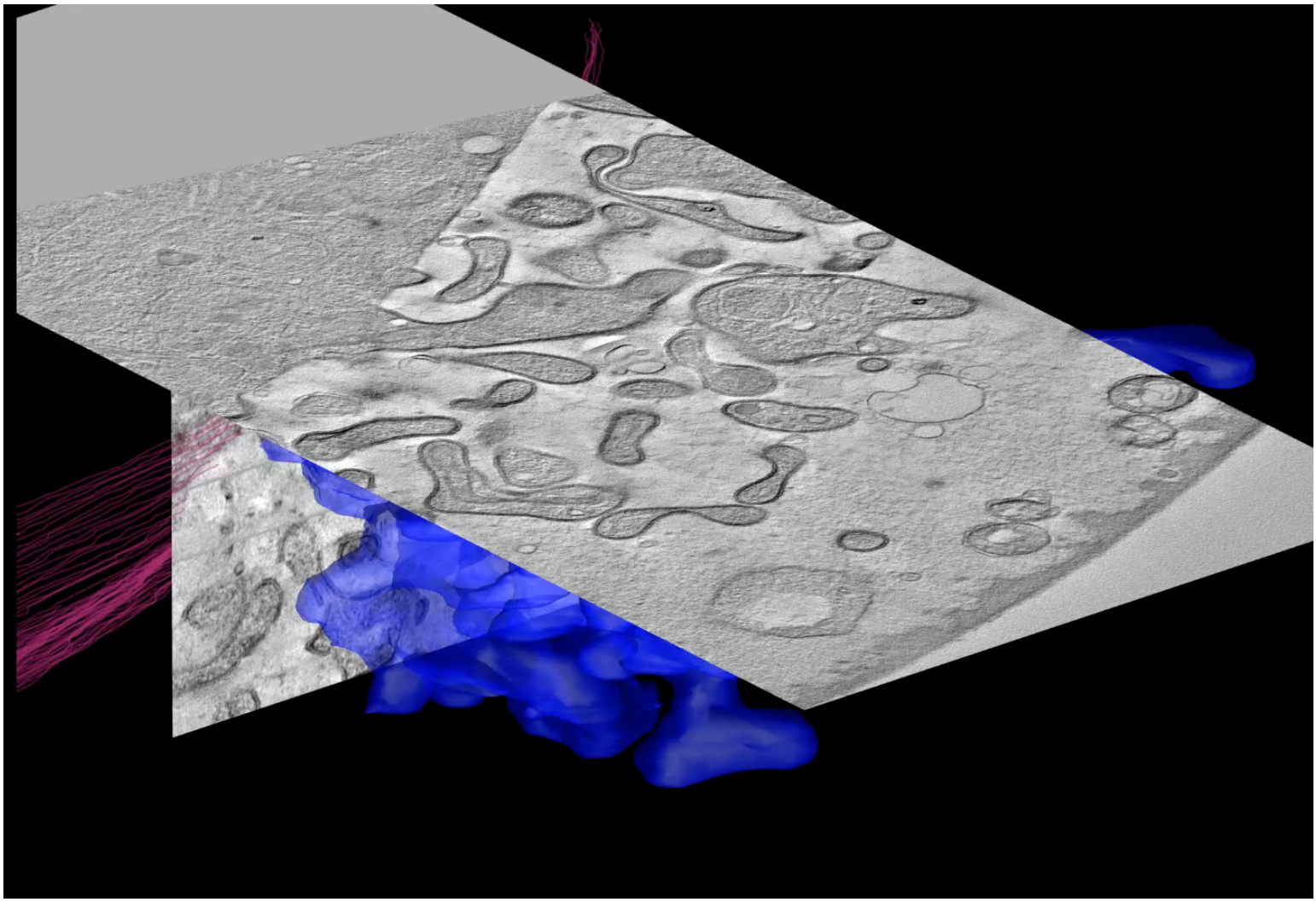

Supplement: Supplementary file 4 — Movie EV2 [file EMBJ-38-e100984-s004.zip › 100984_MovieEV2.pdf]
