# Supplementary material for: Microtubule number and length determine cellular shape and function in Plasmodium
Source: EMBO J. 2019 May 24;38(15):e100984. doi: 10.15252/embj.2018100984 (PMC6669926; doi:10.15252/embj.2018100984)
Supplement: Supplementary file 4 — Movie EV2 [file EMBJ-38-e100984-s004.zip › 100984_MovieEV2.docx]

**Movie EV2 - Tomographic model of a budding *α1-tubulin(-)* sporozoite at day 17 post infection**

3D rendering of 11 serial sections of 300 nm thickness and illustration of the tomographic reconstruction procedure for the plasma membrane (blue) of a budding *α1-tubulin(-)* sporozoite at day 17 post infection. Lines (magenta) indicate the sporoblast membrane from which the parasite buds. Note the multiple connections of the parasite to the sporoblast. Scale bar: 1 µm.
